# Zeolites as Catalysts for Fuels Refining after Indirect Liquefaction Processes

**DOI:** 10.3390/molecules23010115

**Published:** 2018-01-06

**Authors:** Arno de Klerk

**Affiliations:** Department of Chemical and Materials Engineering, University of Alberta, 9211-116th Street, Edmonton, AB T6G 1H9, Canada; deklerk@ualberta.ca; Tel.: +1-780-248-1903

**Keywords:** refining, methanol, Fischer–Tropsch, zeolite catalysts

## Abstract

The use of zeolite catalysts for the refining of products from methanol synthesis and Fisher–Tropsch synthesis was reviewed. The focus was on fuels refining processes and differences in the application to indirect liquefaction products was compared to petroleum, which is often a case of managing different molecules. Processes covered were skeletal isomerisation of *n*-butenes, hydroisomerisation of *n*-butane, aliphatic alkylation, alkene oligomerisation, methanol to hydrocarbons, ethanol and heavier alcohols to hydrocarbons, carbonyls to hydrocarbons, etherification of alkenes with alcohols, light naphtha hydroisomerisation, catalytic naphtha reforming, hydroisomerisation of distillate, hydrocracking and fluid catalytic cracking. The zeolite types that are already industrially used were pointed out, as well as zeolite types that have future promise for specific conversion processes.

## 1. Introduction

Liquid fuels are likely to remain in demand by society for transport applications due to the high energy density and ease distribution of such fuels. One approach to expand the contribution of renewable carbon-sources for use as transport fuel is to employ indirect liquefaction technology followed by fuel refining. The steps involved in the transformation of the renewable carbon-source to liquid transport fuels are synthesis gas production, liquefaction and refining. The liquefaction process determines the nature of the material that must be refined and there are two industrially practiced indirect liquefaction processes, namely, methanol synthesis [1] and Fischer–Tropsch synthesis [2].

There are important differences in the composition of products from indirect liquefaction and that of petroleum [3]. Products from indirect liquefaction are composed entirely of carbon, hydrogen and oxygen, with no nitrogen and sulfur. Products from indirect liquefaction also contain more reactive molecules. There are consequently different molecules to manage during conversion. When petroleum refining technology is employed to refine products from indirect liquefaction, attention must be paid to catalyst selection. The reactivity of the feed material, competitive adsorption, release of water and the presence of organic acids are some of the aspects to consider for catalysis [4]. At the same time the material from indirect liquefaction has more scope for use of acidic zeolites than petroleum derived material, because it is free of nitrogen bases that could suppress activity or deactivate acid catalysts.

The purpose of this work is to provide an overview of refinery processes important for the refining of products from indirect liquefaction and the role of zeolite-based catalysts. To keep the discussion consistent, the zeolites are identified by their framework types [5]. Despite the wide variety of zeolite framework types, only a few zeolite framework types are employed industrially [6]. Nevertheless, the industrial potential of zeolites is considerable, e.g., [7,8]. Key properties of the zeolite framework types that will be encountered in the review are listed in Table 1, with properties taken from [5].

Acid site concentration and acid site strength can be manipulated independent of framework type by changing the composition with the range feasible for the synthesis of that framework type. Different applications of the same zeolite framework type may employ different compositions, e.g., silica-to-alumina ratio, and within the same application there may also be some variations.

The catalysis literature related to Fischer–Tropsch refining was more broadly reviewed before [9]. This paper is restricted to an evaluation of the role of zeolites in acid catalyzed and bifunctional metal-and-acid catalyzed refining processes to produce transport fuels. Refining processes that employ metal catalysis only, such as hydrotreating, will not be discussed unless there is a noted benefit from geometric constraints imposed by a zeolitic support.

## 2. Refining Roadmap

**Figure 1:**
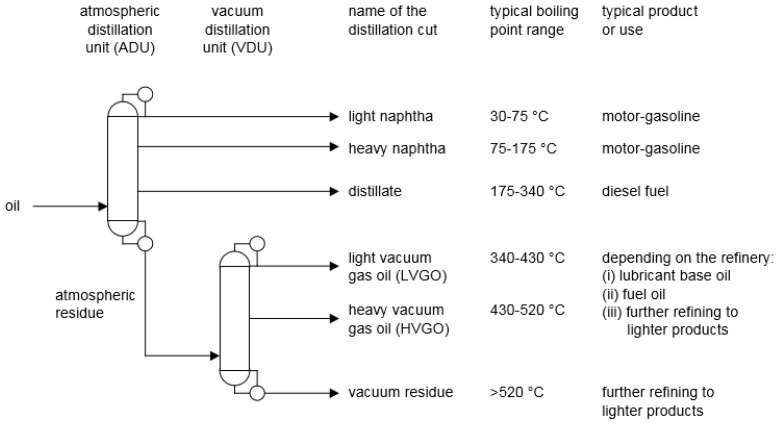
Straight run distillation cuts that are typically produced in an oil refinery.

In a petroleum refinery, the crude oil feed is first distilled into different boiling fractions, or “cuts” (Figure 1).

The material obtained directly from the crude oil, or synthetic crude oil, is called “straight run” to differentiate it from any similar cut produced in the refinery after conversion. Each straight run cut is sent to a refining process that was developed specifically for the conversion of that boiling fraction, which is direct application of the principle of molecular management. The reason for this approach is threefold. First, transport fuels have defined boiling ranges. Second, different transport fuels have different fuel specifications. Third, the molecular composition in each cut is different and depending on the composition there can be significant changes in the reaction chemistry.

The fuel specifications impose molecular requirements on each boiling fraction. It is useful to think of refining as a collection of conversion processes that manipulate the molecular composition of each boiling fraction so that it meets the requirements imposed by the fuel specifications to produce a marketable product. An overview of fuel specifications and how it influences refining can be found in general texts on petroleum refining [10].

For the products from indirect liquefaction to be acceptable as transport fuels, each boiling fraction must meet the same molecular requirements as their petroleum counterparts [11]. When referring to “molecular requirements”, it does not necessarily imply a fixed molecular composition, but rather a collection of molecular properties that imbues the boiling fraction with a desirable macroscopic property, such as density or vapor pressure. Some specifications are more restrictive and specific. For example, the maximum sulfur content or maximum concentration of benzene that will be allowed in the transport fuel.

The discussion of refining processes will be organized in terms of the boiling fractions of the feed materials that must be converted. Typical values for the boiling fractions from different indirect liquefaction processes are given in Table 2 [1,11]. In this respect, the product from methanol synthesis is unique, since it consists mainly of methanol, which is produced with high selectivity. Methanol refining will be dealt with under the category of light oxygenates (Section 4.1). For Fischer–Tropsch products the following broad categories will be used:

(a) Gaseous hydrocarbons. Broadly speaking, gaseous hydrocarbons consist of a mixture of alkanes (paraffins) and alkenes (olefins). Depending on the extent of separation in the Fischer–Tropsch refinery [11], the light hydrocarbons will be present with unconverted synthesis gas and will not be refined. For the purpose of discussion, it will be assumed that the C_3_-C_4_ hydrocarbons are recovered and will be refined. The gaseous hydrocarbons that are produced with crude oil are usually separated from the oil before the oil is transported from the oil well to the refinery, often over great distances. The gaseous hydrocarbons in a petroleum refinery are the products of refining processes and little straight run gaseous hydrocarbons enter a petroleum refinery with the crude oil.
(b) Light oxygenates. The light oxygenates (typically C_1_-C_4_ oxygenates), which include methanol, are water-soluble materials. During condensation, these compounds will dissolve in water that is also condensed after Fischer–Tropsch synthesis. These compounds are unique to indirect liquefaction refineries and no equivalent fraction exists in a petroleum refinery. Methanol and/or ethanol may be imported into petroleum refinery for fuel blending and etherification.
(c) Naphtha. Oil with a boiling range of 30–175 °C (typically C_5_-C_10_ hydrocarbons) is referred to as naphtha. In a Fischer–Tropsch product, the naphtha contains mainly alkanes, alkenes and alcohols, but may also contain aromatics, ketones and carboxylic acids. Petroleum naphtha obtained by distillation from crude oil consists of alkanes, cycloalkanes, aromatics, as well as some sulfur- and nitrogen-containing compounds. In petroleum, naphtha that is produced by conversion processes in the refinery, alkenes may also be present, but alkenes are seldom found in petroleum naphtha obtained by crude oil distillation.
(d) Distillate. Oil with a boiling range of 175–340 °C (typically C_11_-C_22_ hydrocarbons) is referred to as distillate. Apart from boiling point, the composition is similar to that of Fischer–Tropsch naphtha, but the concentration of alkanes is much higher. For petroleum, the same is true, but the concentration of sulfur- and nitrogen-containing compounds is higher than in petroleum naphtha.
(e) Atmospheric residue. Organic products with a boiling point higher than 340 °C are referred to as atmospheric residue. Depending on the type of Fischer–Tropsch technology, this product is either a wax, or an aromatic-rich oil. Atmospheric residues obtained from petroleum distillation reflect the properties of the oil and it can range from a waxy product for paraffinic crude oils to oil with high aromatic content for aromatic crude oils. The concentrations of sulfur- and nitrogen-containing compounds are also dependent on the origin of the oil and the concentration of heteroatom species is higher in the residue than in the lighter fractions. In some oils, there may also be heavy carboxylic acids.

A note for those readers not familiar with refining; boiling ranges are not absolute. There are also many terms to describe the same or slightly different cuts depending on how the cut was produced. Possibly the most confusing of all is that the kerosene cut has a boiling range of 160–260 °C, which overlaps with both the heavy naphtha and distillate boiling fractions. Kerosene is used for the production of jet fuel and is not explicitly shown in Figure 1. Not all refineries are situated close to a jet fuel market and not all fuel refineries produce jet fuel. Jet fuel can also be produced from non-petroleum sources [12], including Fischer–Tropsch synthesis.

## 3. Refining Gaseous Hydrocarbons

### 3.1. Skeletal Isomerisation of n-butenes

**Figure 2:**
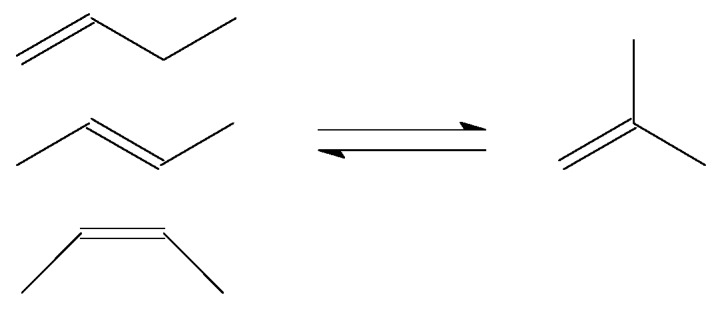
Skeletal isomerisation of *n*-butenes to isobutene.

The production of isobutene from *n*-butenes (Figure 2) may be required as feed preparation for production of blending components for high-octane number motor-gasoline. Two processes that can benefit from isobutene in the feed are etherification and oligomerisation.

Several zeolite types were investigated for this equilibrium limited reaction, but ferrierite (FER) was the only type that was used for industrial production [13]. A key issue in the commercial development of butene skeletal isomerisation was the cycle length that could be obtained due to catalyst deactivation with time on stream [13,14]. Catalyst design had to focus on managing the rate and impact of catalyst coking, by manipulating catalyst morphology, while retaining the FER structure as the active phase [15,16]. It appears that STW might be able to better suppress coking than FER, while delivering the same or better performance as FER [17].

No indirect liquefaction refinery is employing *n*-butenes skeletal isomerisation technology [11].

### 3.2. Hydroisomerisation of n-butane

**Figure 3:**
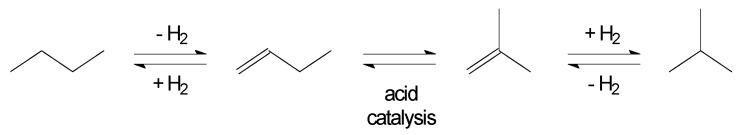
Hydroisomerisation of *n*-butane to isobutane.

Hydroisomerisation of *n*-butane to isobutane (Figure 3) is useful for any refinery with an aliphatic alkylation unit, for which isobutane is a feed material.

The skeletal isomerisation is still performed by acid catalysis. The main difference between hydroisomerisation and skeletal isomerisation is that the concentration of alkenes in the reaction mixture is low to limit side-reactions and it is regulated through the dehydrogenation–hydrogenation equilibrium. There is consequently a need for a dehydrogenation–hydrogenation function on the catalyst and the reaction is conducted in the presence of hydrogen (H_2_). Although there is no net consumption of hydrogen, the hydrogen partial pressure is used to control the dehydrogenation–hydrogenation equilibrium. The dehydrogenation-hydrogenation function is provided by metal promotion and in the case of *n*-butane hydroisomerisation, platinum is used.

The only catalyst type that is in industrial use is platinum-promoted chlorided alumina (Pt/Cl^−^/Al_2_O_3_) [18]. Among competing acidic catalyst supports, this catalyst type is able to perform hydroisomerisation at the lowest temperature. It is an equilibrium limited conversion, where the equilibrium is favored by low temperature, and zeolites are not competitive. It will later be shown that zeolites are used for hydroisomerisation of naphtha (Section 5.1), where they offer better tolerance to feed impurities. The *n*-butane can normally obtained in high purity by pressure distillation and Pt/Cl^−^/Al_2_O_3_ can safely be used, despite its sensitivity to impurities.

This technology is industrially employed for refining indirect liquefaction products [11]. One precaution that is particularly relevant is feed drying. Since all products from indirect liquefaction are produced with some water, care must be taken to remove water to very low levels.

### 3.3. Aliphatic Alkylation

**Figure 4:**
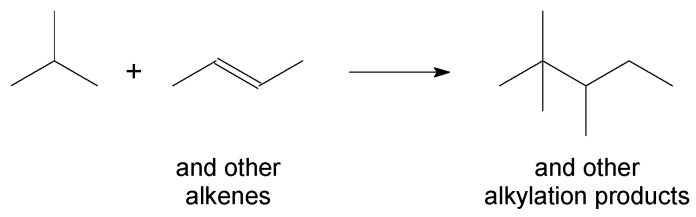
Aliphatic alkylation, illustrated by the reaction of isobutane and *trans*-2-butene. The nature of the alkene will influence the structure of the alkylation product.

Industrial processes for aliphatic alkylation of isobutane with alkenes (Figure 4) to produce high-octane number motor-gasoline blend components employ either HF or H_2_SO_4_ as catalysts.

This does not imply that zeolites and other solid catalysts were not evaluated, but deactivation hampered development leading to an industrial process. The first industrial aliphatic alkylation process to make use of a solid acid catalyst was successfully commissioned only in 2015 [19]. According to the catalysis patent [20] on which the process was based, the solid acid alkylation catalyst is an ultra-stable zeolite Y (FAU) that was promoted with 0.5 wt % platinum. Since then, the catalyst formulation was improved as documented in several subsequent patent applications, e.g., [21]. BEA was also investigated for the same reaction, but was less active and deactivated faster [8].

Aliphatic alkylation technology is industrially used in indirect liquefaction refining [11].

### 3.4. Alkene Oligomerisation

**Figure 5:**
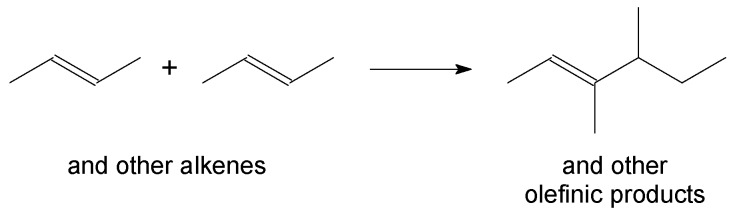
Alkene oligomerisation, illustrated by the dimerisation of *trans*-2-butene.

Alkene oligomerisation, which includes alkene dimerisation (Figure 5), is employed to convert alkenes that are gaseous at ambient conditions into products that are liquids. Catalysts for alkene oligomerisation were recently reviewed by Nicholas [22]. A list of zeolites that form the basis for oligomerisation processes are: MFI, MWW and potentially TON or MFS [6,11].

The choice of catalyst for oligomerisation depends on the application and the type of liquid product that is desired. Different acid catalysts produce liquid products with different degrees of branching and different boiling point distributions. For example, a catalyst that is excellent for producing motor-gasoline would be less well suited for producing diesel fuel. Selectivity, rather than activity, is the deciding factor in selecting an appropriate oligomerisation catalyst.

One aspect that is partly regulated by temperature and partly by geometric constraints is the selectivity to “true oligomers” that have carbon numbers that are integer multiples of the carbon chain length of the alkene feed. It was found that under conditions where both oligomerisation and cracking took place, the ratio of true oligomers to total oligomers was governed by geometric properties of the zeolite. As the ratio of the limiting pore diameter to the largest cavity diameter increased, the ratio of true oligomers to total oligomers increased [23]. The following sequence of increasing true oligomer selectivity was established: FAU < MFI < BEA < TON < MOR [23]. The boiling point distribution is determined by temperature and pressure and becomes independent of the carbon chain length of the feed [24]. At low reaction temperatures, where cracking is not a significant contributor to the reaction network, most oligomers are true oligomers.

Another aspect that is important is the degree of branching, which is the number of branches per oligomer molecule. Again, geometric constraints play a role, with degree of branching increasing in relation to the increase in the area of the pore opening of the zeolite [22]. The following sequence of increasing degree of product branching for a single carbon number product was established: MTT < MFI < MTW [22].

Alkene oligomerisation technology is industrially used in indirect liquefaction refining [11]. The tolerance of MFI for oxygenates is an important attribute. At the PetroSA (formerly Mossgas) Fischer–Tropsch-based gas-to-liquids facility, MFI is used for the conversion of olefins to distillate process [25]. It was also shown that the same MFI catalyst can be employed for oligomerisation using a Fischer–Tropch tail gas that contains unconverted synthesis gas [26].

## 4. Light Oxygenates

### 4.1. Methanol to Hydrocarbons

Methanol is a feed material suitable for both transport fuels production and petrochemical production [1,27,28]. Various technologies were developed around methanol to hydrocarbon conversion, with most of the initial developments centered on MFI as catalyst [29]. The product composition obtained from the conversion of methanol over MFI is similar, but not the same as that obtained by olefin oligomerisation over MFI (Section 3.4). For example, the prevalence of benzene with three or more methyl substituents is higher during methanol to hydrocarbon conversion over MFI than when an olefinic hydrocarbon feed is used.

Methanol itself is a commodity petrochemical and therefore it has a market even without further refining. Much of the recent focus is on using methanol as feed for the production of light alkenes, ethene and propene in particular [30]. For this purpose, it was found that SAPO-34 (CHA) with its very restricted pore geometry had a high selectivity to ethene and propene. Both of these light alkenes are commodity petrochemicals.

Methanol to hydrocarbons can be described as an alcohol dehydration and oligomerisation reaction. One intriguing question is: How are hydrocarbons formed from methanol?

The mechanism of the reaction of methanol to hydrocarbons over MFI and CHA are based on the formation of a carbon pool [31,32]. It appears that trace impurities are responsible for the induction leading to the formation of the carbon pool, after which the hydrocarbon formation and reactions from methanol can be described.

Methanol to hydrocarbon technology was industrially used for fuels production, but presently most methanol to hydrocarbon conversion is aimed at petrochemicals [1].

### 4.2. Ethanol and Heavier Alcohols to Hydrocarbons

**Figure 6:**
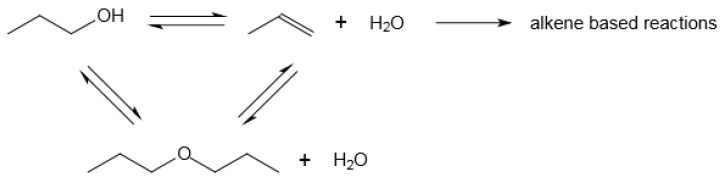
Reaction network of alcohol to hydrocarbon conversion illustrated using 1-propanol; the reaction network shown does not reflect stoichiometry.

The conversion of ethanol and heavier alcohols to hydrocarbons proceeds through well-described steps, with dehydration of the alcohol to form ethers or alkenes as intermediate products. The reaction network is more complicated (Figure 6) and many of the reactions are reversible and equilibrium limited. The alkenes are the hydrocarbon products, but if the catalyst has Brønsted acidity, the alkenes could be further converted by oligomerisation and cracking.

The temperature dependence of the product distribution from alcohol conversion over MFI was illustrated by the work of Gayubo, et al. [33]. Conversion of the alcohols increased as temperature was increased and alkene yield reached a maximum as near complete conversion of the alcohol was achieved. As temperature was further increased, oligomerisation, cracking and aromatisation contributed to the product distribution.

Non-acidic metal oxide catalysts can be used to obtain true dehydration products, where the alkene is of the same carbon structure as the alcohol [34]. Hydrocarbon production for fuels would benefit from the same properties of MFI that makes it useful for methanol to fuels processes (Section 4.1). A comparison of zeolite catalysts for the production of ethene from ethanol, which is the most difficult of the C_2_ and heavier alcohols to dehydrate, obtained the best results with MOR [35]. However, dehydration of heavier alcohols does not require strong Brønsted acidity, nor does it benefit from the imposition of geometric constraints. The value of zeolites in alcohol to hydrocarbon conversion comes from the hydrocarbon conversion that is taking place after dehydration.

Conversion of ethanol and heavier alcohols to alkenes has historically been one of the routes for producing alkenes [36]. However, C_2_ and heavier alcohol dehydration is not practiced in Fischer–Tropsch refineries.

### 4.3. Carbonyls to Hydrocarbons

**Figure 7:**
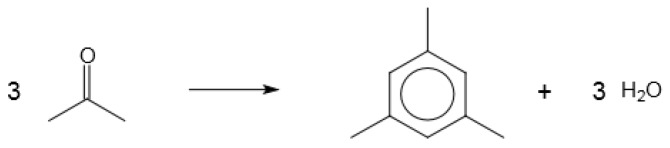
Conversion of carbonyl compounds to aromatic hydrocarbons, illustrated by the reaction of acetone to form mesitylene (1,3,5-trimethylbenzene).

Carbonyl compounds, ketones and aldehydes, can be converted over acid catalysts to aromatic hydrocarbons (Figure 7). The mechanism involves stepwise aldol condensation and dehydration that eventually leads to the formation of an aromatic [37]. For example, it is possible to produce benzene from ethanal (acetaldehyde) and 1,3,5-trimethylbenzene from propanone (acetone) or propanal.

In addition to a variety of non-zeolitic catalysts [38], the aromatisation of carbonyl compounds was investigated over MFI [37] and FAU [39] zeolites. The reaction selectivity and operating conditions are influenced by the nature of the acid catalyst, but the chemistry required for the reaction also proceeds in bulk solution in the presence of an acid catalyst.

Since the primary product is aromatic, catalyst deactivation by coking is an issue to consider in selecting a catalyst. It may also be possible to affect product selectivity when using zeolites, because the primary aromatic products are radially symmetric and bulky in nature, with the exception ethanal to benzene conversion. Transalkylation that is influenced by the geometric constraints of a zeolite catalyst might be useful when converting a mixture of C_2_-C_4_ carbonyl compounds, as is found in Fischer–Tropsch aqueous products.

Carbonyl to aromatic hydrocarbon conversion is not industrially employed.

### 4.4. Etherification of Alkenes with Alcohols

**Figure 8:**
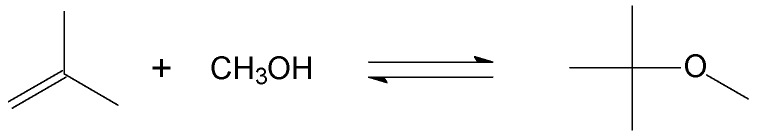
Etherification of alkenes illustrated by the reaction of isobutene and methanol to produce methyl *tert*-butyl ether (MTBE).

The etherification of isobutene and isopentene (2-methyl-1-butene and 2-methyl-2-butene) with either methanol or ethanol produces fuel ethers (Figure 8).

Fuel ethers are high octane number blending components used in motor-gasoline blending that do not have some of the drawbacks of direct alcohol blending [10]. The use of fuel ethers fell into disfavor and is prohibited in some regions. Since alcohols are not present in petroleum, alcohols had to be imported into petroleum refineries employing an etherification process.

Etherification is an equilibrium limited reaction and the forward reaction is favored by lower temperature. The preferred catalyst type for etherification is acidic resin catalysts, e.g., [40,41]. It is possible to perform the conversion to near complete equilibrium conversion at temperatures below 100 °C. Nevertheless, some studies performing etherification over zeolites, such as MFI, FAU and BEA, can be found in the literature [42,43].

Alkene etherification is performed industrially on indirect liquefaction products using acidic resin catalysts. Two different applications are found, namely, fuel ether production for motor-gasoline and the use of etherification as part of linear 1-alkene production processes to produce linear 1-alkenes as petrochemicals used as co-monomers in polymerisation [11].

## 5. Naphtha

### 5.1. Hydroisomerisation of Light Naphtha

The hydroisomerisation of light straight run naphtha, which is rich in *n*-pentane and *n*-hexane, is practiced in many refineries that produce high octane number motor-gasoline. The octane number of the linear hydrocarbons can be improved by hydroisomerisation to produce branched products. The catalysis is the same as for the hydroisomerisation of *n*-butane to isobutane (Section 3.2). The mechanism of hydroisomerisation over the zeolite Pt/MOR was discussed in detail in literature [44].

Unlike *n*-butane, the light naphtha is a feed material that is more likely to contain some contaminants. Process selection is determined by the composition of the feed, and the commercial catalyst offerings are therefore more varied (Table 3) [45,46].

Lower temperature benefits the isomerisation equilibrium by favoring branched products. The main advantage of using Pt/MOR, is its higher tolerance of feed contaminants. In an indirect liquefaction environment, water tolerance is very important. The light naphtha is seldom free of low amounts of oxygenates that will produce water on hydrogenation over Pt. The ability of Pt/MOR to tolerate more C_7_ hydrocarbons in the feed is an indirect measure of the coking-resistance when processing a feed that is more prone to cracking and producing alkenes. This attribute is also important. The straight light run naphtha from Fischer–Tropsch synthesis is rich in alkenes. It was demonstrated that 1-pentene can be used as feed for both hydrogenation and hydroisomerisation over Pt/MOR as long as the process is engineered appropriately to manage the additional hydrogen consumption and heat release during reaction [47].

Current industrial implementation of light naphtha hydroisomerisation in Fischer–Tropsch refining is a two-step process, whereby the light naphtha is first hydrotreated in a naphtha hydrotreater and then hydroisomerised [11]. By doing so, the light naphtha is essentially free of oxygenates, water and alkenes; it is therefore possible to make use of Pt/Cl^−^/Al_2_O_3_ catalyzed hydroisomerisation.

### 5.2. Catalytic Naphtha Reforming

Catalytic naphtha reforming is a key refining unit in the production of motor-gasoline. Heavy naphtha, which has a low octane number, is converted into an aromatic-rich product that has a high octane number. In addition to this, hydrogen is produced. In many conventional petroleum refineries, this is the only or primary source of H_2_. The H_2_ is needed for hydrotreating, making the catalytic naphtha reformer an important unit, not only due to its role to improve the octane number of the heavy naphtha, but also due to the production of H_2_ for the refinery.

The catalyst type universally used for catalytic naphtha reforming to produce motor-gasoline is noble metal promoted chlorided alumina, e.g., Pt/Cl^−^/Al_2_O_3_ [10]. Conversion is performed at high temperature and the pressure is kept as low as practical, to limit catalyst deactivation. There is an inherent volume loss associated with the process due to the increase in density of the product, as well as the formation of some gaseous hydrocarbons due to acid cracking on the catalyst. For a given feed material the yield decreases as the octane number of the product increases.

The yield versus octane number performance of Pt/Cl^−^/Al_2_O_3_-type catalysts during catalytic naphtha reforming is strongly dependent on the content of cyclic material that is present in the heavy naphtha. This is a consequence of the catalysis. Conversion of cyclohexanes to high octane number aromatics requires only dehydrogenation. Conversion of alkylcyclopentanes to high octane number aromatics requires only isomerisation to cyclohexanes and then dehydrogenation. The most difficult material in the feed to convert to high octane number products is *n*-alkanes. This presents a challenge for catalytic naphtha reforming of Fischer–Tropsch-derived heavy naphtha, which contains a high percentage of *n*-alkanes [11].

**Figure 9:**
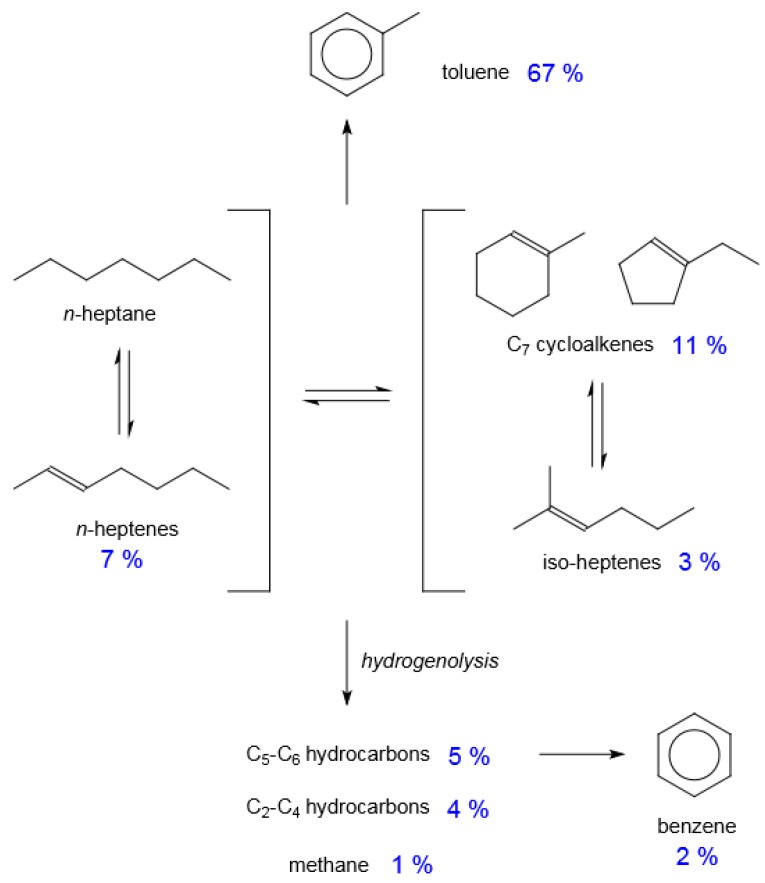
Reaction network of catalysis over Pt/K/LTL illustrated with selectivity values obtained at 86% *n*-heptane conversion at 500 °C, near atmospheric pressure, weight hourly space velocity of 0.68 h^−1^ and C_7_:H_2_ feed rate of 1:6.

Platinum promoted non-acidic L-zeolite (LTL) was found to be very efficient for the conversion of C_6_ and longer chain *n*-alkanes to aromatics. To illustrate this, the reaction network is shown (Figure 9) together with selectivity data from literature [48]. The main catalysis pathways are dehydrogenation, dehydrocyclisation and hydrogenolysis.

The high selectivity of *n*-alkane to aromatics conversion was originally ascribed to molecular orientation that facilitated terminal adsorption of *n*-alkanes and *n*-hexane in particular [49]. Other explanations were also forwarded and were reviewed [50]. The most likely explanation appears to be control of entry into the cavities of the LTL by the narrow pore mouth, which reduces the probability of bimolecular reactions taking place and also suppresses deactivation by coke formation [51]. Despite the selectivity of Pt/K/LTL catalysts for *n*-alkane conversion to aromatics, there is some hydrogenolysis by Pt. Recent work on suppressing hydrogenolysis in Pt/K/LTL catalysts include addition of low amounts of Ag [52], use of secondary mesoporosity [53], and both lowering Pt content and controlling the Pt cluster size [48].

Process technology was developed using Pt/K/LTL catalysts for refining with a petrochemical focus. One of the main challenges applying this technology to petroleum refining was the remarkable sulfur sensitivity of the Pt/K/LTL catalyst [50]. In this respect Fischer–Tropsch heavy naphtha is a good feed material, because it is sulfur-free, as long as upstream naphtha hydrotreating does not employ a sulfided hydrotreating catalyst.

Existing refineries based on Fischer–Tropsch technology that produce on-specification transport fuels, employ conventional catalytic naphtha reforming technology based on Pt/Cl^−^/Al_2_O_3_-type catalysts [11]. Despite the potential benefit to switch to Pt/K/LTL catalysts, such a conversion is not simple, because the upstream naphtha hydrotreaters employ sulfided hydrotreating catalysts and sulfur is introduced to maintain these catalysts in sulfided state.

## 6. Distillate

### Hydroisomerisation of Distillate

Straight run distillate refining is normally limited to hydrotreating and sweetening. The cold flow properties of petroleum derived distillate are usually within specification for jet fuel and diesel fuel, unless the distillate is obtained from a very paraffinic crude oil. The distillate from Fischer–Tropsch synthesis is very paraffinic and it has a high linear hydrocarbon content. It is therefore not surprising that the distillates produced from industrial Fischer–Tropsch processes, even after refining, have cold filter plugging point temperatures in the range –4 to –7 °C [2]. Diesel fuels produced from such distillates are suitable for use only in warmer climates.

Hydroisomerisation is required to reduce the freezing point of kerosene for jet fuel production and to improve the cold flow properties of the overall distillate for diesel fuel production for colder climates. The bifunctional catalysis needed for hydroisomerisation of distillate is the same as that described in Section 3.2 and Section 5.1, but with one important feed related difference. Distillate range material typically has a carbon chain length of C_11_ to C_22_, which makes the molecules larger and more prone to acid catalyzed cracking than light naphtha [54]. In this respect, the hydroisomerisation of distillate has similar catalyst design requirements as found in hydroisomerisation catalysts for the production of lubricant base oils from vacuum gas oil cuts. The catalysis challenge is to isomerise the *n*-alkanes to branched alkanes, without significant cracking of the branched alkanes.

If the loss of some of the distillate to lighter products can be tolerated, hydrocracking catalysts based on MFI that are industrially used for catalytic dewaxing can be considered [55]. In order to achieve only hydroisomerisation, the first generation of vacuum gas oil hydroisomerisation catalysts were based on amorphous acidic supports, such as amorphous silica-alumina and fluorided alumina [55]. The ability of reaction products to diffuse out of the catalyst after hydroisomerisation was found to be important to suppress secondary reactions, such as hydrocracking [56,57]. When suppression of hydrocracking was desirable, good results at a high *n*-alkane conversion were reported for model and industrial feed materials with platinum promoted SAPO-11 (AEL) [58].

The hydroisomerisation of indirect liquefaction distillate is not industrially practiced, partly due to the locations where the products are sold, and partly due to the impact of overall fuel blending. When the ratio of hydrotreated straight run distillate is small compared to the total volume of distillate that is produced, hydroisomerisation of the distillate may not be needed. The blending of straight run distillated with distillate from alkene oligomerisation (Section 3.4) and/or hydrocracking of atmospheric residue (Section 7.1) that is more branched in nature, dilutes the deleterious impact of the *n*-alkane content of the straight run distillate on the cold flow properties.

## 7. Atmospheric Residue

### 7.1. Hydrocracking

**Figure 10:**
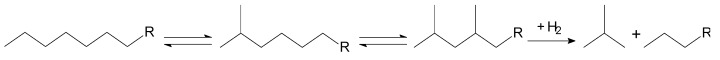
Hydroisomerisation and hydrocracking of *n*-alkanes. Isomerisation and cracking steps take place by dehydrogenation, acid catalysis and hydrogenation, which are not explicitly shown.

Hydrocracking catalysts are also hydroisomerisation and hydrotreating catalysts (Figure 10). The conversion employs dehydrogenation-hydrogenation equilibrium to maintain a low alkene concentration, which also results in hydrogenation of the cracked products. Thus, converting atmospheric residue by hydrocracking causes the product to be more isomerised, more hydrogenated and have a lower boiling point distribution.

The distillation range and extent of product isomerisation is determined by the nature of the hydrocracking catalyst in combination with the operating conditions employed. The extent of product isomerisation is affected by the ratio of hydrogenation–dehydrogenation activity (metal function of catalyst) to the isomerisation and cracking activity (acid function of catalyst). When the metal function activity is high compared to the acid function activity, the product tends to be more branched [59]. In the case of waxes, the carbon chain length of the feed affects the extent of isomerisation of the product [60]. Catalyst accessibility is also an important consideration when dealing with large molecules and it is not surprising that commercial zeolite-based hydrocracking catalysts for petroleum are all based on FAU due to its large pore size [61]. These are general statements about hydrocracking.

The potential role of zeolites for modifying the product distribution is related to shape selectivity caused by the specific structure of each zeolite type. Reactant, product, and transition state selectivity can all be used to limit further conversion of isomerised products, or to limit the formation of isomerised products that are more prone to cracking [56]. The interaction of linear molecules with zeolites is also affected by length-dependent phenomena, such as the “window”-effect [62,63]. The latter is particularly important as potential tool for manipulating the carbon number distribution of products from wax hydrocracking, which is otherwise of equal random probability along the chain, except for the three carbon bonds on either side of the molecule.

Wax is a special feed case when it comes to hydrocracking. There might be potential for other zeolite types to achieve specific benefits during wax hydrocracking, some of which have already been mentioned in relation to hydroisomerisation (Section 6) over zeolites, such as MFI and AEL. In addition to those already mentioned, several metal-promoted zeolite types were evaluated for *n*-alkane hydrocracking, among other BEA, MOR, and TON [57,64]. However, a sobering assessment of the potential for different zeolite types to improve hydrocracking performance, without sacrificing catalyst stability was made by Rigutto [61]; the promise of wider-pore zeolites was recognized, but it was pointed out that thus far no clear benefit over FAU could be demonstrated.

Wax hydrocracking is a key refining technology for indirect liquefaction based on low temperature Fischer–Tropsch synthesis and it is industrially employed in several facilities [11]. The products from wax hydrocracking are naphtha and distillate range products consisting of a mixture of linear and branched alkanes, with distillate being the preferred product from hydrocracking. Different facilities employ different hydrocracking catalysts for wax based on amorphous acidic supports.

The only zeolite-based hydrocracking catalyst used with indirect liquefaction products is MFI that is used for dewaxing the atmospheric residue fraction from high temperature Fischer–Tropsch synthesis [11].

### 7.2. Fluid Catalytic Cracking

Conversion of vacuum gas oil and vacuum residue by fluid catalytic cracking (FCC) relies on a combination of thermal decomposition at high temperature and acid catalyzed cracking. As was pointed out [65], not all cracking in fluid catalytic cracking should be attributed to catalytic cracking.

The products from fluid catalytic cracking are more olefinic and aromatic in nature and it is a technology that is primarily used to produce naphtha range products for motor-gasoline and light alkenes for petrochemicals. Various process technologies are available [18], and the choice of technology and catalyst depends on the feed quality and the preferred products. In petroleum refineries close to petrochemical markets light alkenes, propene in particular, is normally a desirable product due to its higher value than that of transport fuel.

The main zeolite type used in fluid catalytic cracking is FAU [66]. The success of ultrastable Y zeolites in fluid catalytic cracking is partly due to the accessibility of FAU for large molecules. The term ultrastable Y zeolite, refers to FAU where the silica-to-alumina ratio that is greater than 6:1, which is usually achieved through dealumination by steaming [67]. By decreasing the alumina content, the acid site density is reduced. When the acid site density is reduced, hydrogen transfer reactions are suppressed, which leads to an increase the alkene content of the product and decrease the formation of aromatics and alkanes [66]. Another common modification is the exchange of FAU with rare earth metal oxides to improve activity and stability [68]. The most commonly used rare earth metal oxides are La_2_O_3_ and CeO_2_ [68]. Exchange with other materials has also been investigated, e.g., [69]. Light alkene production can be increased through more severe operation (higher temperature), as well as by using MFI as catalyst [70]. When MFI is used to increase propene production, it is usually used in conjunction with FAU, with FAU still performing most of the vacuum gas oil and residue cracking to lighter products.

Coke is inevitably formed during fluid catalytic cracking and a moderate amount of coke is desirable from a process point of view. Coke combustion during catalyst regeneration provides the heat necessary for the catalytic cracking to proceed. To quote Magee and Dolbear [71]: “It is a truism that the unit’s operation must be optimized around heat balance as a primary restraint, and that catalyst and feedstock choices are made to operate within this constraint.” In this respect, the use of a more paraffinic feed material poses a challenge, because it has a low coke yield even at high conversion [72].

Nevertheless, over the years several studies reported on the fluid catalytic cracking of wax over FAU, MFI and BEA catalysts [72,73,74,75,76]. It is commonly noted that high wax conversion is possible leading to high C_3_-C_4_ gas yields, low coke yield and low aromatic content. This is consistent with what one would expect from the cracking of a high hydrogen-to-carbon ratio material. As with petroleum, fluid catalytic cracking of wax employing MFI leads to more propene than over the other zeolite catalysts.

Fluid catalytic cracking of Fischer–Tropsch wax is not practiced industrially, although there is one facility that employs fluid catalytic cracking of Fischer–Tropsch naphtha at high riser outlet temperature using MFI to boost propene production [11]. To compensate for the lack of coking of the catalyst, due to the hydrogen-rich nature of the light feed, additional fuel has to be injected in the regeneration section to achieve heat balance.

## 8. Discussion and Conclusions

Methanol synthesis and Fischer–Tropsch synthesis processes produce products that can make use of conventional refining technologies, but that benefit from the modification of technologies and selection of different catalysts. An exception is oxygenate conversion technologies that are not normally found in petroleum refineries. For both indirect liquefaction processes, technologies that are specific to oxygenate conversion and are tolerant for water extend the range of conversion processes that are useful for refining. In this respect MFI (ZSM-5) and CHA (SAPO-34) are industrially used with demonstrated benefit.

Having reviewed indirect liquefaction refining processes in Section 3, Section 4, Section 5, Section 6 and Section 7, a list of the processes that could benefit from zeolite catalysts were compiled (Table 4).

Not all of the opportunities for the use of zeolite catalysts are industrially exploited. Yet, there are a number of hydrocarbon conversion processes that stand out as instances where the appropriate selection of a zeolite catalyst for use with indirect liquefaction products could in future lead to more efficient conversion and/or improve on current refinery designs.

(a) Catalytic naphtha reforming using Pt/K/LTL zeolite catalysts benefit from the high linear hydrocarbon content and sulfur-free nature of indirect liquefaction products. Although this type of catalyst is associated predominantly with petrochemical production, its potential benefit for fuel refinery design is clear [77], particularly for converting *n*-heptane, which is a challenging molecule to refine for motor-gasoline.
(b) Hydroisomerisation of distillate using Pt/AEL (SAPO-11) zeolite catalysts has the potential to increase the yield of jet fuel from Fischer–Tropsch refineries. It may also become a necessary addition to refineries that want to market Fischer–Tropsch-derived diesel fuels in cold climates without relying on blending with petroleum derived distillate.

Zeolite-based catalysts for wax hydrocracking hold promise, but there is not a clear zeolite type, or clear benefit derived from a specific zeolite type that stands out at present. However, sulfur-free wax with a high *n*-alkane content is an ‘ideal’ feed for hydrocracking. Ways to manipulate hydroisomerisation in relation to hydrocracking with zeolite-based catalysts have been described. However, one challenge that remains and where zeolite catalysts that exploit the window effect might provide a solution in the future, is to regulate cracking position along the hydrocarbon chain. Hydrocracked distillate has desirable properties for jet fuel and diesel fuel, but hydrocracked naphtha requires further extensive refining to have desirable properties for motor-gasoline. A zeolite that could regulate cracking position would constitute a breakthrough in hydrocracking catalysis.

## Figures and Tables

**Table 1:** Zeolite framework types and properties relevant to fuels refining processes.

Framework Type	Type Used in Refining	Ring Size/atomsunit	Channel Size/nm × nm
AEL	SAPO-11	10	0.65 × 0.40
CHA	SAPO-34	8	0.38 × 0.38
FAU	Y-zeolite	12	0.74 × 0.74
FER	Ferrierite	8 and 10	0.35 × 0.48 and 0.42 × 0.54
LTL	L-zeolite	12	0.71 ×0.71
MFI	ZSM-5	10 and 10	0.51 × 0.55 and 0.53 × 0.56
MFS	ZSM-57	8 and 10	0.33 × 0.48 and 0.51 × 0.54
MOR	Mordenite	8, 8 and 12	0.26 × 0.57, 0.34 × 0.48 and 0.65 × 0.70
MWW	MCM-22	10 and 10	0.40 × 0.55 and 0.41 × 0.51
TON	ZSM-22	10	0.46 × 0.57

**Table 2:** Typical product distribution from different indirect liquefaction processes.

Product	Product Distribution/wt %		
	Methanol Synthesis	Low Temperature Fischer-Tropsch Synthesis ^a^	High Temperature Fischer-Tropsch Synthesis
*gaseous hydrocarbons*			
methane	1	4	13
ethane/ethene	-	2	10
C_3_-C_4_ hydrocarbons	-	8	24
*light oxygenates*			
C_1_-C_4_ oxygenates	99	4	10
*oil products*			
light naphtha	-	4	15
heavy naphtha	-	8	18
distillate	-	20	7
atmospheric residue	-	50 ^b^	3 ^c^

^a^ Typical industrial Fe-based catalyst. Co-based catalysts have a slightly different distribution; ^b^ Waxy product; ^c^ Aromatic-rich oil.

**Table 3:** Summary of commercial light naphtha hydroisomerisation catalysts.

Description	Pt/Cl/Al_2_O_3_	Pt/SO_4_^2−^/ZrO_2_	Pt/MOR
Operating temperature (°C)	130–150	180–210	250–280
Water tolerance (µg/g)	0	20	200
Sulfur tolerance (µg/g)	0	20	200
C_7_ hydrocarbon tolerance (%)	2	2	5

**Table 4:** Summary of the refining technologies relevant to indirect liquefaction where zeolite catalysts could be preferred catalysts.

Technology	Zeolites	Comments
*Gaseous hydrocarbons*		
skeletal isomerisation of *n*-butenes	FER	performance limited by rate of coking
hydroisomerisation of *n*-butane	-	catalyst selection related to feed purity
aliphatic alkylation	FAU	new industrial process; metal promoted
alkene oligomerisation	MFI, MWW, TON, MFS	product target drives catalyst selection
*Light oxygenates*		
methanol to hydrocarbons	MFI, CHA	MFI for fuels, CHA for light alkenes
C_2_+ alcohols to hydrocarbons	-	
carbonyls to aromatic hydrocarbons	-	
etherification of alkenes with alcohols	-	
*Naphtha*		
hydroisomerisation of light naphtha	MOR	more tolerant to feed impurities
catalytic naphtha reforming	LTL	high selectivity for C_6_/C_7_ *n*-alkanes
*Distillate*		
hydroisomerisation of distillate	AEL	less hydrocracking at high conversion
*Atmospheric residue*		
hydrocracking	AEL, MFI	amorphous supports used for wax
fluid catalytic cracking	FAU, MFI	FAU standard, MFI to boost propene
